# Safety in nuclear power plants in India

**DOI:** 10.4103/0019-5278.44693

**Published:** 2008-12

**Authors:** R. Deolalikar

**Affiliations:** Occupational Health, Narora Atomic Power Station Hospital, Type C, 11/2, N.A.P.S. Colony, N.A.P.P. Narora, Bulandshahr, Narora - 202 389, Uttar Pradesh, India

**Keywords:** Atomic energy act, atomic energy regulatory board, dose limits, emergency planning and measures, environmental radiological surveillance, epidemiological survey, nuclear facilities, nuclear power plants, radioactive waste management, radiological protection, safety, the disaster management act, zoning concept

## Abstract

Safety in nuclear power plants (NPPs) in India is a very important topic and it is necessary to dissipate correct information to all the readers and the public at large. In this article, I have briefly described how the safety in our NPPs is maintained. Safety is accorded overriding priority in all the activities. NPPs in India are not only safe but are also well regulated, have proper radiological protection of workers and the public, regular surveillance, dosimetry, approved standard operating and maintenance procedures, a well-defined waste management methodology, proper well documented and periodically rehearsed emergency preparedness and disaster management plans. The NPPs have occupational health policies covering periodic medical examinations, dosimetry and bioassay and are backed-up by fully equipped Personnel Decontamination Centers manned by doctors qualified in Occupational and Industrial Health. All the operating plants are ISO 14001 and IS 18001 certified plants. The Nuclear Power Corporation of India Limited today has 17 operating plants and five plants under construction, and our scientists and engineers are fully geared to take up many more in order to meet the national requirements.

## INTRODUCTION

Safety in nuclear power plants (NPPs) is often less understood and more talked about and, thus, I wanted to share the facts with the readers. With reference to an article published in an earlier issue of this journal,[1] it became all the more pertinent to clear the myths.

At the very start, may I state that any discussions on the Indo–US deal are outside the purview of this article. I would like to focus only on the safety aspects of the NPPs in India.

Currently, all the NPPs in India are under the Nuclear Power Corporation of India Limited (NPCIL). Hence, any discussions regarding the NPPs in India will pertain to it. The NPPs in India are not only safe but are also well regulated, have proper radiological protection of workers and the public, regular surveillance, dosimetry, approved standard operating and maintenance procedures, a well-defined waste management methodology, proper well documented and periodically rehearsed emergency preparedness and disaster management plans. The NPPs have occupational health policies covering periodic medical examinations, dosimetry and bioassay and are backed-up by fully equipped Personnel Decontamination Centers manned by doctors qualified in Occupational and Industrial Health. Moreover, they have specialized training in handling radiological emergencies.

Safety in NPPs in India is a very vast subject and would need reams of papers to cover it aptly. However, I have tried to summarize it to the best possible level. I hope that I would succeed in making the reader understand the magnanimity with which these plants are operated and that they are entirely safe.

Safety is accorded overriding priority in all the activities. All nuclear facilities are sited, designed, constructed, commissioned and operated in accordance with strict quality and safety standards. Principles of defense in depth, redundancy and diversity are followed in the design of all nuclear facilities and their systems/components. The regulatory framework in the country is robust, with the independent Atomic Energy Regulatory Board (AERB) having powers to frame the policies, laying down safety standards and requirements and monitoring and enforcing all the safety provisions. The AERB exercises the regulatory control through a stage-wise system of licensing. As a result, India's safety record has been excellent in over 277 reactor years of operation of power reactors.

Nuclear power generation is governed by a legislation, the Atomic Energy Act, 1962. The Atomic Energy Act encompasses all the activities concerned with atomic energy, including electricity generation.

## RADIOLOGICAL PROTECTION OF WORKERS[2–16]

Radiological protection of the workers is ensured by the following measures:

### Design aspects

The design considerations that have a bearing on radiation protection in NPPs include:

1. Proper design, plant layout and adequate shielding:
  Design values are prescribed for the radiation level at a specified distance from the equipment/components as well as for the general radiation fields in different areas of the plant. The plant layout is such that the areas are segregated according to their radiation levels and contamination potential. The design, layout of areas and equipment, maintenance approach and shielding, etc. are made such that the collective dose to the station personnel would be “as low as reasonably achievable” (ALARA) and meet the specified regulation on collective dose.
2. Limits of air contamination levels in different zones of the plant:
  Provision of ventilation is made such that in full-time occupancy areas of the plant, the airborne contamination are maintained below 1/10 Derived Air Concentration.
3. Source control by proper selection of materials/components:
  Materials used in plant systems are selected in such a way that the activation products arising from the base material or the impurity content do not significantly contribute to radiation exposures.
4. Design limit for collective dose:

A limit on the collective dose is specified at the design stage of each NPP so that adequate provisions for radiation protection are made in the design of the plant to keep radiation levels in different areas below design levels.

### Dose limits

The AERB has prescribed the following dose limits for exposures to ionizing radiations for occupational workers:

1. Effective dose (whole body)
  1.1 Twenty Milli- Sievert (mSv)/year averaged over five consecutive years, calculated on a sliding scale of 5 years. (The cumulative effective dose in the same 5-year period shall not exceed 100 mSv.)
  1.2 A maximum of 30 mSv in any year.
2. Equivalent dose (individual organs)
  2.1 Eye lens 150 mSv/year.
  2.2 Skin 500 mSv/year.
  2.3 Extremities 500 mSv/year (hands and feet).
3. Pregnant woman
  3.1 Equivalent dose limit to the surface of the woman's lower abdomen (for the remaining period of pregnancy) – 2 mSv.
  3.2 Annual Limit on Intake (ALI) for radionuclides – 0.05 ALI. (For the remaining period of pregnancy.)
4. Apprentices and students (above the age of 16 years)
  Effective dose (whole body): 6 mSv/year.
  Equivalent dose (individual organs)
  4.1 Eye lens 15 mSv/year.
  4.2 Skin 50 mSv/year.
  4.3 Extremities 50 mSv/year (hands and feet).

In addition, investigation limits are also prescribed by AERB at which investigation of exposure cases exceeding these limits are carried out by an AERB committee.

Effective dose means summation of the tissue equivalent doses, each multiplied by the appropriate tissue-weighting factor.

Sliding scale of 5 years means the current year and the previous 4 years.

Average dose over 1 cm^2^ of the most highly irradiated area of the skin.

For temporary workers, separate control limits, lower than that for regular workers, are prescribed.

The external and internal exposures of all the plant personnel are assessed on a monthly basis. For assessing the internal dose in Pressurized Heavy Water Reactors, a bioassay program on a weekly basis and a dose estimation software are used. A computerized dose data management system is used, which helps in updating the data for effective dose control.

### Organization in radiation protection

Each NPP has a Health Physics Unit (HPU), comprising of a group of trained and experienced radiation protection professionals, who, in coordination with plant management, implement the radiation protection program in the plant. The HPUs in all NPPs in the country are entrusted with the responsibility of providing radiological surveillance and safety support functions. These include monitoring of areas, personnel, systems, effluents, exposure control and exposure investigations. The HPUs are part of the Bhabha Atomic Research Center (BARC) and are independent of the NPP organization, and have direct channels of communication with the top plant management in enforcing the radiation protection program.

**Figure 1:**
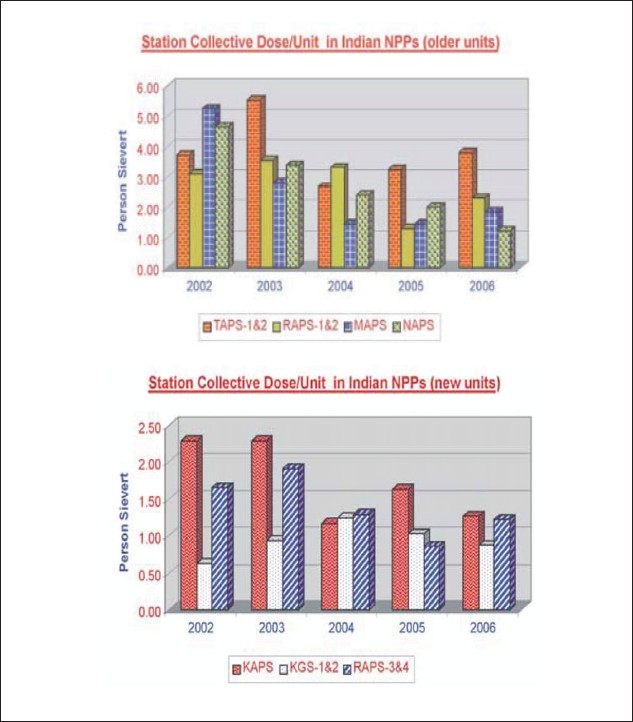
Station Collective Dose / Unit in Indian NPPs – Old and New Units

The individual and the collective dose consumed in the plant is reviewed in detail and measures for reduction are devised at the plant level. These measures include engineering and administrative solutions such as shielding, ventilation, use of protective equipment, procedure adherence, work permit system, access control, display of placards, job planning, mock up, training, supervision, etc. In addition, a three-tier arrangement is in place to review and monitor implementation of recommendations pertaining to radiological safety. The first level review is carried out at the plant and the regulatory body performs the second- and third-levels reviews. There has been no case of annual exposure exceeding 20 mSv during the last 3 years in all the NPPs. The collective annual dose to plant personnel is kept below the annual dose budget approved by the AERB. Efforts are made each year to reduce this progressively [Figure 1].

### Steps for ALARA exposures

In order to meet the objectives of the ALARA, working procedures and methods are examined with regard to the possibility of reducing doses resulting from radiation jobs. During the operational stage of the NPP, the exposures are kept ALARA by implementing the following operational aspects:

1. Implementation of radiation protection and contamination control procedures.
2. Use of proper protective equipment.
3. Adherence to approved operating and maintenance procedures.
4. Implementation of radiation protection training and qualification programs and conduct of refresher courses to impart ALARA concept and awareness.
5. Proper work planning and its practical implementation through the issuance of Radiation Work Permit and dose budgeting for each operation.
6. Constitution of an ALARA committee at the plant level.
7. Planning and preparedness for unusual events.

### Radiation protection review by AERB

The Atomic Energy (radiation protection) Rules, 2004, form the basis of regulatory control activities related to radiation protection. These rules are implemented by the utilities through various procedures. In addition, the AERB practices other measures to exercise control on radiation protection aspects for NPPs, which, among others, include the following:

1. Collective dose budget
  The AERB approves the annual collective dose budget for each NPP. The stations are required to propose the budget along with planned activities. The AERB committees review the collective dose expenditure and the proposed budget and, based on the review, formally approve the annual budget within which all the operation and maintenance activities have to be managed.
2. Review of excess exposure cases
  Exposure cases exceeding the investigation limits are investigated and reported by the Exposure Investigation Committee set up at each NPP. Such reports are reviewed by the AERB Safety Committees and the Safety Review Committee for Operating Plants. The root causes of such exposures are established and corrective measures are recommended.
3. Regulatory inspection
  The adequacy of the radiation protection program and its implementation in the operating NPP are inspected twice a year. The deficiencies are reported and corrective measures are recommended and followed-up through enforcement procedures.
4. Review of the radiological safety aspects
  A regulatory body reviews the report on radiological safety aspects of the plant on a quarterly and annual basis.

## RADIOLOGICAL PROTECTION OF PUBLIC

The following measures ensure the radiological protection of the public due to the operation of a NPP.

### Design aspects

1. Dose limits for members of the public
  The sources contributing to generation of radioactive solid, liquid and gaseous wastes and their release to the environment are examined with respect to minimization of waste at the source at the design stage itself. The dose to public resulting from these releases are assessed and, if necessary, appropriate design measures to reduce these releases are introduced.
2. Exposure criteria for accident analysis
  The design analysis should demonstrate that the calculated doses to the members of the public at the site boundary under design basis accident condition should not exceed the reference doses prescribed by the AERB.

### Dose limit

The AERB has prescribed the following limits to a member of the public at exclusion distance due to releases of radioactive effluents from nuclear facilities at a site:.

**Figure 2:**
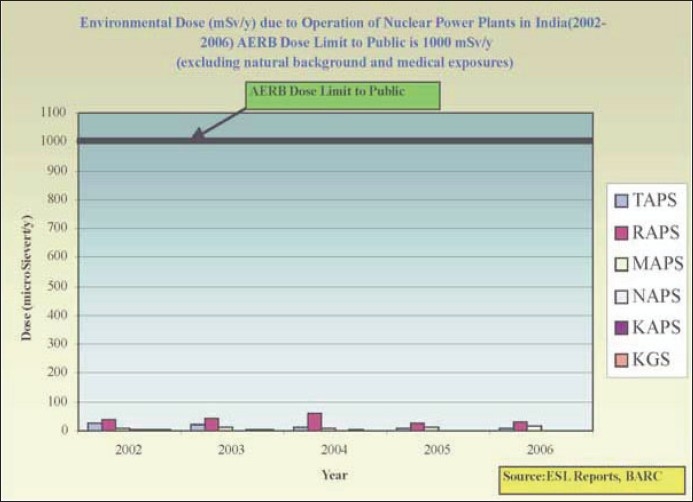
Environmental Dose due to Operation of NPPs in India

Effective dose (whole body): 1 mSv (1000 *μ*Sv)/year. The Figure 2 shows the environmental dose from various NPPs in India over the period 2002–2006, which is less than 10% of the prescribed limits.

Equivalent dose (individual organs)

1. Eye lens 15 mSv/year.
2. Skin 50 mSv/year.

## RADIOACTIVE WASTE MANAGEMENT

The performance of the radioactive waste management system established at NPPs is reviewed to ensure that appropriate methods and management practices continue to be in place and the generation of radioactive waste is kept to as minimum as practicable in terms of activity and volume.

1. Method of disposal and monitoring
  Gaseous wastes from reactor buildings are filtered using prefilters and high-efficiency particulate air filters and released after monitoring through a stack of 100 m height. The release rate and integrated releases of different radionuclides are monitored and accounted for to demonstrate that the releases are within the prescribed limits.
  The radioactive liquid wastes generated in a NPP are segregated, filtered and conditioned as per procedure and after adequate dilution to comply with the limits of discharges, disposed to the environment water body. The activity discharged is monitored at the point of discharge and accounted on a daily basis. The AERB has prescribed limits on the annual volume and activity of discharge, daily discharges and activity concentration from each NPP, which are site specific. The radioactive solid wastes are disposed off in brick-lined earthen trenches, Re-enforced cement concrete (RCC) vaults or tile holes, depending on the radioactivity content and the radiation levels.
2. Authorized limits of discharge
  The discharge of radioactive waste from a NPP is governed by the Atomic Energy (safe disposal of radioactive wastes) Rules, 1987, which is issued under the Atomic Energy Act, 1962. It is mandatory for a NPP to obtain authorization under the above rules from the competent authority for disposal of radioactive wastes.
  The regulatory limits (authorized limits) of radioactive effluents are based on the apportionment of an effective dose limit of 1 mSv/year to public arising from nuclear facilities at a site considering all the routes of discharges and significant radionuclides in each route of discharge. Derived limits corresponding to the dose apportioned for different radionuclides are established taking into account the site-specific parameters.
  Authorized limits are set at a much lower value than derived limits to achieve effluent releases ALARA. The releases from NPPs have been only a fraction of release limits specified.

## ENVIRONMENTAL RADIOLOGICAL SURVEILLANCE

An elaborate environmental survey program around each NPP site is carried out by the HPUs and the Environmental Survey Laboratories (ESLs) of BARC. The basic objective of the environmental monitoring and surveillance program is to assess the radiological impact under all states of the NPP and demonstrate compliance with the radiation exposure limits set for the members of the public by the AERB. This is achieved by carrying out a radiological surveillance of the environment by professionals of the ESLs. The HPUs and ESLs are part of the BARC and are independent of the utilities. They provide the regulatory body with periodic reports on radiological conditions of the NPPs and the results of environmental surveillance. The ESL is established several years before operation of a NPP. Extensive surveys are carried out around each nuclear power station to collect data on the dietary intake. During the preoperational phase, the annual intake of cereals, pulses, vegetables, fish, meat, eggs and milk are established by direct survey. Elaborate studies of the topography of the site, land use pattern and population distributions are carried out systematically during the preoperational phase. Also, a detailed epidemiological survey of the population is carried out in the preoperational phase. This is carried out by a neutral agency like the Tata Memorial Hospital or any closely located University Medical College. Along with the topographical and dietary studies, the ESL also carries out the work of establishing the preoperational background radiation levels. Extensive micrometeorological data such as wind speed and wind direction, temperature and rain fall are collected for a few years to identify the worst sector and critical population. The ESL continues its monitoring and surveillance program during the operation phase of the NPP. The samples for analysis are selected on the basis of potential pathways of exposure. The program undergoes modification based on experience. Generally, more samples are collected near the vicinity of the plant and from locations where population clusters exist, and the sampling frequency reduces with the distance. Areas up to a distance of 30 km are covered under the environmental survey program. Although the main emphasis is on samples that are relevant directly to the estimation of the dose, such as drinking water, edible food items, air, etc., a number of other samples are also assayed for radioactivity and used as trend indicators. From the radioactivity level in the environmental matrices, intake parameters and dose conversion factors, the population dose is estimated. The annual effective dose to members of the public in the vicinity of the NPPs have been estimated by ESLs and found to be only a few *μ*Sv. ESLs are accredited laboratories that take part in inter comparison studies conducted by the International Atomic Energy Agency.

## EMERGENCY PREPAREDNESS

### General

NPPs are designed, constructed, commissioned and operated in conformity with existing stringent nuclear safety standards. These standards ensure an adequate margin of safety so that NPPs can be operated without undue radiological risks to the plant personnel and members of the public. Notwithstanding these safety standards, it is necessary to develop, as a measure of abundant caution and in conformity with international practices, emergency response plans so that any eventuality, howsoever unlikely, with a potential to result in undue radiological risk to plant personnel and public, is handled effectively. The preparedness and response to emergencies are important responsibilities of the operating organization. All NPPs have established and documented emergency procedures by having an on-site emergency preparedness plan. Similarly, the plan with respect to off-site emergency is made available with the district authority. The role, responsibilities and action plans for various agencies required to act during an emergency are detailed in these plans.

### National laws, regulations and requirements

The Government of India has enacted “The Disaster Management Act, 2005”, which provides for the effective management of disasters, including accidents involving NPPs. As per the provisions of this act, the National Disaster Management Authority (NDMA) has been established at the national level, whose chairperson is the Prime Minister. The NDMA has the responsibility for laying down policies, plans and guidelines for disaster management for ensuring timely and effective response to any disaster. In line with the above national plan, a state plan and district plans are drawn up by the respective authorities constituted for the purpose.

Specific requirements with respect to emergency preparedness in NPPs have been formulated by the AERB in the various regulations, as given in references.

### Zoning concept and emergency planning

In India, a NPP is generally sited in a relatively low-population zone, with the basic objective of limiting the dose-received members of the public and population as a whole under normal and accident conditions to ALARA levels. In order to achieve the above objective, the area around the NPP is divided into the following zones:

1. Exclusion zone
  An exclusion zone of 1.5 km radius around the plant is established, which is under the exclusive control of the operating organization, and no public habitation is permitted in the area. The dose limits to a member of the public, under normal operating conditions and under design basis accident conditions specified, are applied at the boundary of this exclusion zone.
2. Sterilized zone
  With the help of administrative measures, efforts are made to establish a sterilized zone up to a 5-km radius around the plant. This is the annulus around the exclusion zone, which has the potential for extensive contamination in case of a severe accident. Development activities within this area are controlled so as to check an uncontrolled increase in the population. In this area, only natural growth of the population is permitted.
3. Emergency planning zone (EPZ)
  This is the zone defined around the plant up to a 16-km radius and provides for the basic geographical framework for decision making on implementing measures as part of a graded response in the event of an off-site emergency. The EPZ is examined in great detail while drawing up an offsite emergency plan and arranging logistics for the same. The entire EPZ is divided into 16 equal sectors. The objective is to optimize the emergency response mechanism and to provide the maximum attention and relief to the regions most affected during an offsite emergency.

### Emergency measures

The emergency measures consist of emergency actions in respect of notification, alerting personnel, assessment of situation, corrective actions, mitigation, protection and control of contamination. These are detailed in the emergency response manual.

1. Notification
  Any emergency situation will be promptly notified to the concerned personnel as per the notification plan. The message conveyed in the notification is required to be clear and concise.
2. Assessment action during emergency
  Indicating, recording and annunciating instruments provided in the main control room, radiation surveys, environmental surveys, meteorological data and status of plant are utilized to assess the situation and to predict the projected doses. These assessment actions enable planning timely corrective and protective actions.
3. Corrective actions
  These actions are taken to correct plant abnormal situations and to bring the plant under control. The types of corrective actions are decided by the situations prevailing at that point of time.
4. Protective measures (countermeasures)
  These actions are taken to mitigate the consequences of a radiological event and to protect site personnel, members of the public and livestock from radiation. These include sheltering, administration of prophylactics, control on consumption of contaminated foodstuff and, finally, evacuation. It is essential to ensure that the response measures would reduce the overall impact to the public to a level significantly lower than what they would be in the absence of such measures. The emergency response manual gives details of the protective measures and the intervention levels approved by the AERB for initiating protective measures to limit radiation exposures. Evacuation is a very effective countermeasure but is very carefully considered before a decision to implement is taken. The benefits and risks of this countermeasure are carefully assessed in terms of averted dose. If radiation levels in the affected zone continue to exist beyond acceptable levels, then relocating the affected population is resorted to.
5. Contamination control measures
  Contamination control measures are meant to check the spread of radioactive contamination. These actions include segregation of highly contaminated persons and decontaminating them, decontamination of vehicles, regulating the traffic, access control to prevent unauthorized entry to keep traffic routes open solely from the emergency response point of view, confiscation of contaminated food stuff and substituting fresh uncontaminated food in its place, banning fishing in contaminated sea/river water, banning the consumption of contaminated water and its replacement with contamination-free water, identification of contaminated areas requiring excavation and disposal of contaminated soil, decontamination of contaminated dwellings or their disposal and destroying the contaminated crops and grass.
  I hope that this comprehensive piece of information would go a long way in making the reader understand how meticulously our NPPs operate and how much of efforts, infrastructure and man power are directed solely in catering to the safety of the personnel and the public. All the operating plants are ISO 14001 and IS 18001 certified plants.
  Before I end, I would assure all the readers that the NPPs in India are very much in safe hands. The NPCIL today has 17 operating plants and five plants under construction, and our scientists and engineers are fully geared to take up many more in order to meet the national requirements.
